# Hepatosplenic Abscesses and Osteomyelitis of the Spine in an Immunocompetent Adult with Cat Scratch Disease

**DOI:** 10.1155/2015/317260

**Published:** 2015-10-21

**Authors:** D. Knafl, F. Lötsch, H. Burgmann, G. Goliasch, W. Poeppl, M. Ramharter, F. Thalhammer, C. Schuster

**Affiliations:** ^1^Division of Infectious Diseases and Tropical Medicine, Department of Internal Medicine I, Medical University of Vienna, 1090 Vienna, Austria; ^2^Division of Cardiology, Department of Internal Medicine II, Medical University of Vienna, 1090 Vienna, Austria; ^3^Division of Immunology, Allergy and Infectious Diseases, Department of Dermatology, Medical University of Vienna, 1090 Vienna, Austria

## Abstract

We present an 18-year-old, immunocompetent Austrian military conscript with cervical lymphadenopathy, fever, back-pain, and persistent inflammation markers despite two weeks of antimicrobial therapy with ampicillin/sulbactam. All specific laboratory investigations for identification of a specific etiology, including blood cultures and autoantibodies, were inconspicuous. Abdominal computed tomography showed multiple hypodense hepatosplenic lesions and osteomyelitis of the thoracic and lumbar spine with base plate fracture. Based on the patient's history, clinical presentation, and radiological findings, serology for cat scratch disease (CSD) was performed and high *B. henselae* specific IgM and IgG antibodies were detected. Due to its variety of clinical presentations, diagnosis of CSD is challenging, especially in the absence of a history of specific exposure. This case report shall remind the physician that cat scratch disease is a common disease, mainly presenting with fever and lymphadenopathy in young patients. Nevertheless CSD has many different and rare forms of presentations, including hepatosplenic lesions and bone involvement as shown in this case.

## 1. Introduction

*Bartonella henselae* (*B. henselae*) is a Gram-negative bacterium that belongs to the alpha2-subgroup of the class Proteobacteria. Two serotypes are known: Houston-1 serotype and Marseille serotype [1]. Its major vector is the cat flea (*Ctenocephalides felis*) [2, 3]. Transmission to humans occurs via contaminated cats' claws and teeth, which gives it the name cat scratch disease. Normally* Bartonella* rapidly migrates from the blood into endothelial cells. There it causes vasculoproliferative lesions: by activation of inflammatory and proinflammatory cascades, it stimulates angiogenesis and the formation of new capillaries from old ones, leading to bacillary angiomatosis and bacillary peliosis [4, 5]. Bacillary peliosis is a rare condition caused by* B. henselae* infections of the parenchymal vasculature, which results in development of cystic, blood filled spaces in the liver, spleen, bone marrow, or lymph nodes, but furthermore it can also cause abscess-like formations [5].

Bone involvement is a rare phenomenon in CSD. The infection probably spreads via the haematogenous route, accounting for disseminated cases, and via the lymphatic route, for cases with limited extension [6]. It is most frequently reported in children and young adults and normally presents as fever with lymphadenopathy in the draining site of a cat scratch or bite [7].

## 2. Presentation

In early November 2014 an 18-year-old man presented to our department with fever above 40°C, headaches, night sweats, and diffuse arthralgia for the last two weeks. Suspecting a bacterial infection with an unknown focus, he had been treated with ampicillin/sulbactam in a secondary care hospital for two weeks.

The patient reported having been on a one-week holiday in Turkey three months ago. At the time of admission he was doing his military service. Before military service he lived at his parents' farm, where several different animal species are kept, including cattle, chicken, and cats. The full medical examination showed no abnormalities except bilateral cervical lymphadenopathy.

Laboratory investigations revealed markedly elevated C-reactive protein (CRP) of 11.41 mg/dL on day of admission. White blood count was within the normal range with 9.21 G/L. Differential blood count showed a decrease in lymphocyte count of 16%.

Antimicrobial therapy was stopped and serial blood cultures, serologies for* Entamoeba*,* Francisella tularensis*,* Brucella* spp., and fungal, bacterial, and viral broad-spectrum PCRs were performed. All results were negative and did not provide evidence for a specific infectious pathogen. Similarly, QuantiFeron-TB test for* Mycobacterium tuberculosis* showed negative results and screening for autoimmune antibodies including rheumatoid factor, c-ANCA, and p-ANCA did not indicate autoimmune disease.

**Figure 1:**
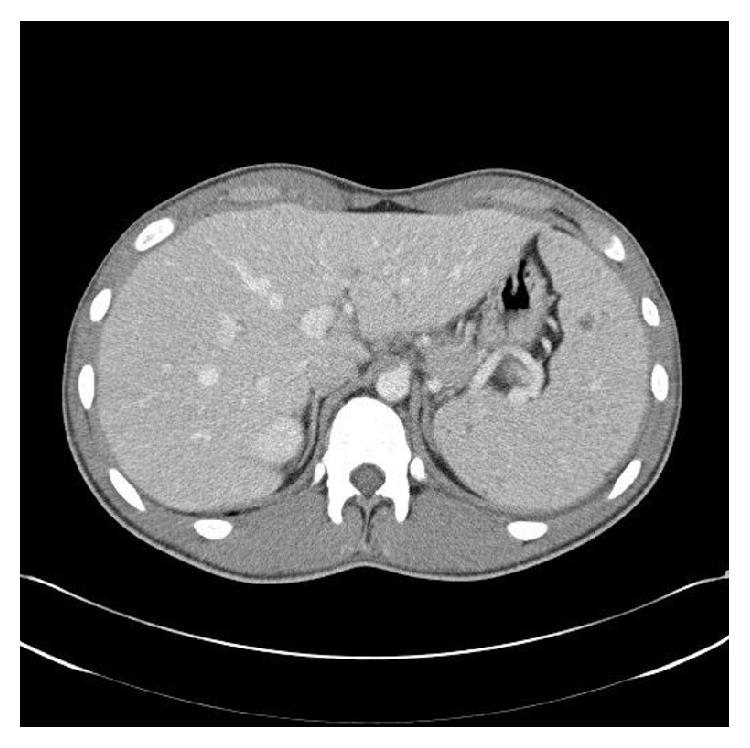
Computer tomography (CT) of the chest and abdomen. Transverse views showed multiple hepatic and splenic lesions.

Due to the patient's symptoms, such as fever and lymphadenopathy, and his inconclusive laboratory findings, a CT scan was performed, which showed multiple hypodense hepatic and splenic lesions; the largest is 2 cm in diameter (Figure 1). Furthermore subchondral sclerosis and erosion of the ventral thoracic spine were shown with maximal affection of the seventh vertebral body.

**Figure 2:**
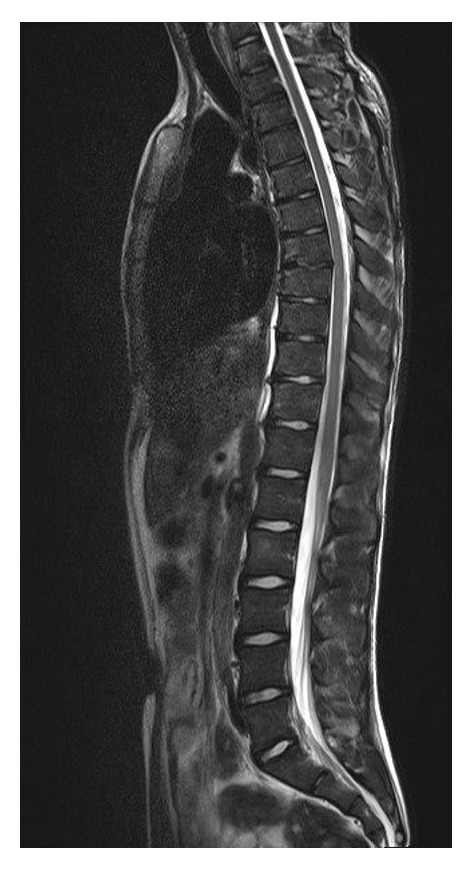
MRI of the spine, showing a fracture of the base plate in thoracic spine body 7, such as lesions in lumbar spine body 2.

**Figure 3:**
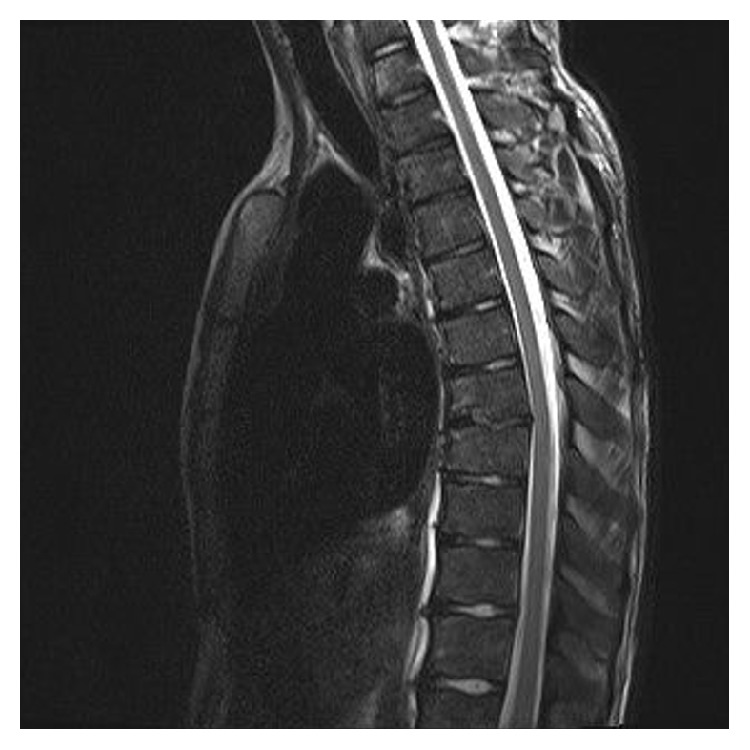
MRI of the spine showing the fracture of the base plate in thoracic spine body 7.

**Figure 4:**
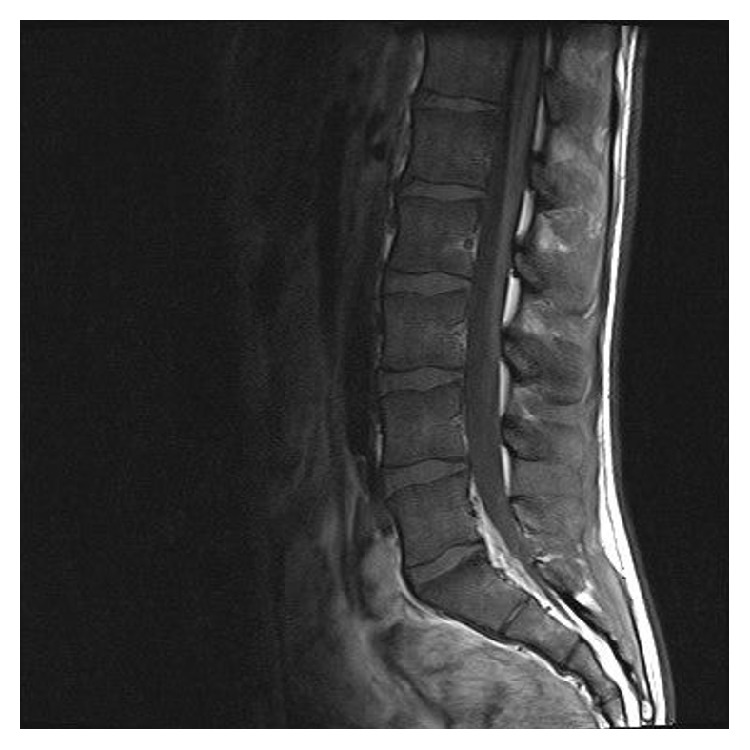
MRI of the spine showing lesions in lumbar spine body 2.

Given the ambiguous structure of the spine, a MRI was done, confirming a ventral base plate fracture of vertebral body 7 with reduction in height of 20% and bone marrow edema (Figures 2, 3, and 4). The lesions were suggestive for an underlying infectious origin as were the hepatosplenic lesions.

Due to the radiological appearance, the stagnant infection parameters with negative serologies for* Francisella tularensis*,* Brucella* spp.,* Entamoeba*, and blood cultures for common infectious pathogens, and the patient's history of residency on a farm with cats, serologic testing for* B. henselae* was performed. IgM (1 : 1000) and IgG (1 : 10.000) antibodies for* B. henselae* were highly elevated. Consequently, according to recently published guidelines [8], the diagnosis of an atypical presentation of visceral cat scratch disease with bone involvement was established.

Consequently, regarding a histopathological examination of a cervical lymph node, we decided not to take a biopsy, to avoid potential risks involved with this procedure, especially since diagnosis could be established with the patient's clinical presentation, history, laboratory findings, and serology. A PCR for* Bartonella henselae* could not be performed, as this test is not available in our hospital.

## 3. Treatment and Follow-Up

Intravenous antimicrobial treatment with azithromycin (1.5 g total dose) and rifampicin 300 mg twice daily for 3 weeks was administered and resulted in a rapid decrease of inflammation markers (CRP of 11.4 mg/dL to 1.32 mg/dL within one week).

Further follow-up serology for* B. henselae* remained positive with IgM antibodies 1 : 1000 two weeks after discharge. CRP was decreasing to 0.81 mg/dL at this point.

**Figure 5:**
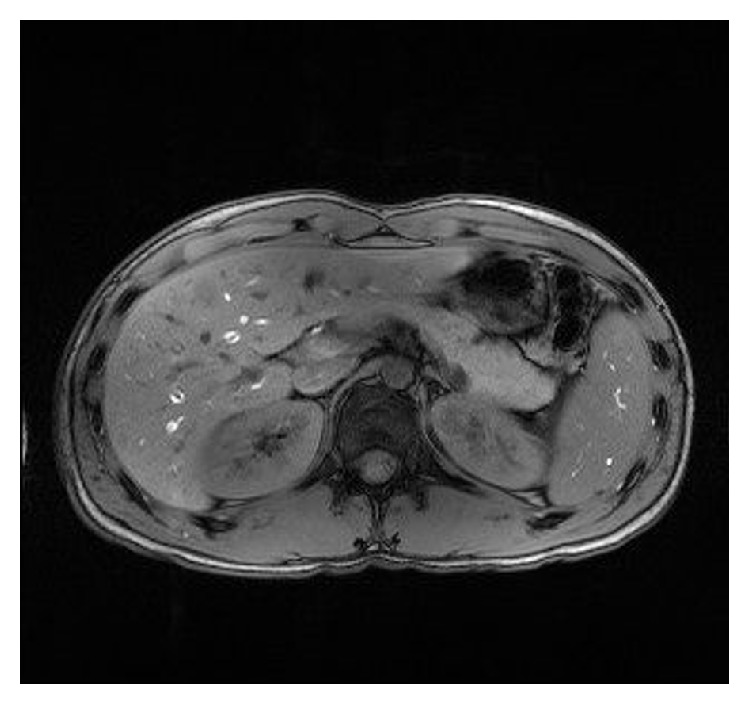
Follow-up MRI with most likely fluid-filled hepatic and splenic lesions.

**Figure 6:**
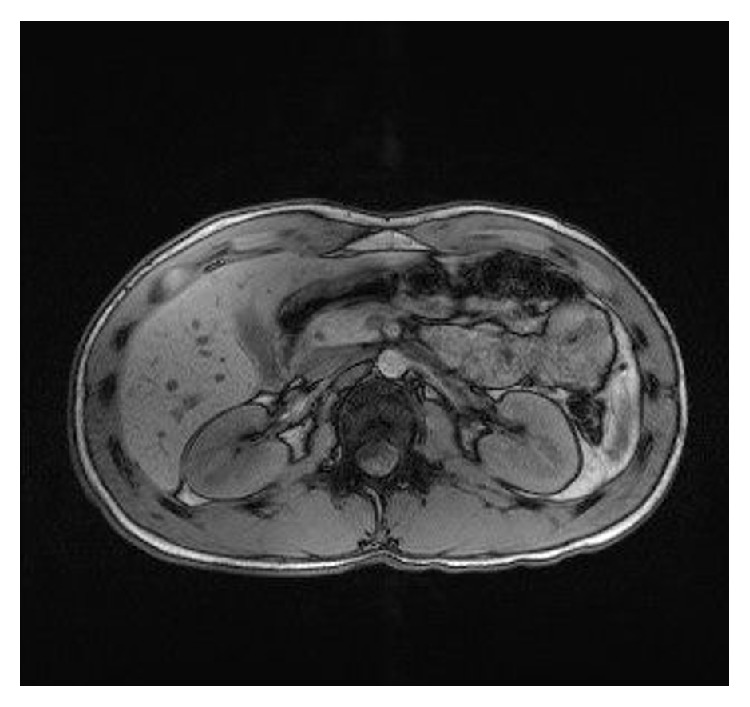
Follow-up MRI with fluid-filled hepatic lesions.

One month after initiation of antimicrobial therapy, abdominal MRI revealed markedly reduced hepatic lesions with a maximum of 1.2 cm in diameter (Figures 5 and 6).

## 4. Differential Diagnosis

Diagnosis of CSD is often challenging, as the pathogen is very slowly growing and therefore hardly detectable in blood cultures. Histopathology usually shows a granulomatous infection with an acellular, necrotic center and* B. henselae* can be identified with Warthin-Starry silver stain. Nevertheless, even in histopathological examination the diagnosis of* B. henselae* is often challenging. Only recently, PCR for* B. henselae* has become available in clinical routine; however sensitivity is often low. Therefore* B. henselae* specific serology is still the main diagnostic tool in clinical practice.

Indirect fluorescence assay (IFA) and enzyme immunosorbent assay (EIA) are the main diagnostic features in detection of* B. henselae*. Both show high sensitivity (88%) and high specificity (97%) [6, 9–16]. But still, there is significant cross-reaction with* B. quintana* and in patients with recent EBV infection (IgM positive for EBV), IgM for* B. henselae* might be wrongly positive. If a recent infection with* B. henselae* is suspected in a patient with IgM antibodies against EBV, physicians are advised to check on elevated IgG antibodies for* B. henselae*. If those are elevated as well, the patient is highly suspicious for being infected with CSD [17].

Nevertheless Brucellosis and Tularemia should be considered as differential diagnosis for patients presenting with symptoms as described above. Although both pathogens are very rare in Central Europe, even among military personnel, there should be a serologic testing for Tularemia and Brucellosis [18]. Both diseases are zoonotic infections with a broad range of symptoms and should definitely be excluded before diagnosis of CSD can be established; therefore at least serologies for theses pathogens should be performed if specific PCRs should not be available.

## 5. Discussion

This case report shows a very rare presentation of CSD and how difficult and time-consuming diagnosis may be. Only 10% of patients with* B. henselae* infection develop hepatic granuloma or splenic abscess [8, 19]. Similarly, bone involvement is a rare phenomenon and only 0.27% of patients reportedly develop osteomyelitis. Similar to our case, bone localization most often affected is the vertebral column (42%), followed by the pelvic girdle (27%) [6].

As suggested by Margileth we agree that, to establish the diagnosis of CSD, three of four of the following criteria should be present [20]:

- Cat or flea contact regardless of the presence of an inoculation site lesion.
- Negative serology for other causes of lymphadenopathy; sterile pus aspirated from a node; a positive* Bartonella* PCR assay; and/or liver or spleen lesions seen on CT scan.
- Positive serology for* B. henselae* with a titer ratio of ≥1 : 64.
- Biopsy showing granulomatous inflammation consistent with CSD or a positive Warthin-Starry silver stain.

This report underlines the importance of considering CSD as differential diagnosis in young patients with fever of unknown origin and lymphadenopathy [21]. Furthermore this case illustrates that CSD may present atypically and that* B. henselae* specific serology may provide the decisive information to reliable establish the diagnosis of CSD.
